# The Application of Deep Learning to Accurately Identify the Dimensions of Spinal Canal and Intervertebral Foramen as Evaluated by the IoU Index

**DOI:** 10.3390/bioengineering11100981

**Published:** 2024-09-29

**Authors:** Chih-Ying Wu, Wei-Chang Yeh, Shiaw-Meng Chang, Che-Wei Hsu, Zi-Jie Lin

**Affiliations:** 1 Department of Neurosurgery, China Medical University Hsinchu Hospital, Hsinchu 302, Taiwan; zingwu1029@gmail.com; 2 Engineering Management, National Tsing Hua University, Hsinchu 300044, Taiwan; shiii96206@gmail.com (C.-W.H.); a03398978@gmail.com (Z.-J.L.)

**Keywords:** artificial intelligence, deep learning, magnetic resonance image, vertebral foramen, intervertebral canal

## Abstract

Artificial intelligence has garnered significant attention in recent years as a rapidly advancing field of computer technology. With the continual advancement of computer hardware, deep learning has made breakthrough developments within the realm of artificial intelligence. Over the past few years, applying deep learning architecture in medicine and industrial anomaly inspection has significantly contributed to solving numerous challenges related to efficiency and accuracy. For excellent results in radiological, pathological, endoscopic, ultrasonic, and biochemical examinations, this paper utilizes deep learning combined with image processing to identify spinal canal and vertebral foramen dimensions. In existing research, technologies such as corrosion and expansion in magnetic resonance image (MRI) processing have also strengthened the accuracy of results. Indicators such as area and Intersection over Union (IoU) are also provided for assessment. Among them, the mean Average Precision (mAP) for identifying intervertebral foramen (IVF) and intervertebral disc (IVD) through YOLOv4 is 95.6%. Resnet50 mixing U-Net was employed to identify the spinal canal and intervertebral foramen and achieved IoU scores of 79.11% and 80.89%.

## 1. Introduction

The lumbar spine is a complex anatomical structure composed of bony vertebrae, intervertebral joints, ligamentum flavum, and intervertebral discs. These elements work in concert with the paraspinal muscles to maintain the overall stability of the human body, facilitating both static posture and dynamic trunk movements. The lumbar spinal canal and intervertebral foramina provide critical protection for the dura mater and nerve roots, which are essential for motor functions of the lower limbs as well as the regulation of urinary and fecal excretion [1]. Degenerative lumbar spine disease (DLSD) is a major health problem worldwide, considered a leading cause of disability, and an issue worthy of discussion, with an estimated 266 million individuals affected globally each year [2]. DLSD includes a spectrum of conditions, such as intervertebral disc herniation, spondylolisthesis, spinal stenosis, and intervertebral foraminal stenosis. These conditions lead to lumbar instability and nerve root compression. This can clinically present as low back pain, neurogenic claudication, radiculopathy, or, in severe cases, cauda equina syndrome [3]. To accurately diagnose DLSD, clinicians typically rely on various imaging modalities, including X-rays, Magnetic Resonance Imaging (MRI), and computed tomography (CT). The choice of imaging technique is guided by the patient’s specific symptoms, clinical signs, and neurological examination findings. Among these modalities, MRI is particularly valuable due to its high sensitivity in detecting abnormalities in lumbar soft tissues, intervertebral foramina, the ligamentum flavum, discs, and cerebrospinal fluid [4]. MRI enables the precise identification of lumbar spine lesions, allowing for prompt and appropriate intervention. However, despite the diagnostic power of MRI, interpretation can be subject to human error and variability.

Over the past twenty years, Artificial Intelligence (AI) advancements have dramatically transformed medical practice, addressing limitations in disease diagnosis, precision medicine, medical imaging, error reduction, and healthcare cost efficiency [5,6,7]. AI technologies and intense learning algorithms have shown promise in enhancing the accuracy and consistency of diagnoses, especially in complex cases involving multiple potential pain generators such as low back pain and sciatica. The development and application of AI in analyzing spinal imaging studies, including MRI, represent a growing area of interest, offering potential improvements in diagnostic precision and patient outcomes [8,9,10,11]. The spinal canal and intervertebral foraminal stenosis are key anatomical factors that contribute to the pathophysiology of low back pain and sciatica. Recognizing the importance of accurately diagnosing these conditions, we have developed a deep learning (DL) architecture specifically designed to evaluate and analyze cross-sectional images of the spinal canal and intervertebral foramina in the lumbar spine. This model aims to enhance the diagnostic process by reducing human bias and increasing the accuracy of detecting clinically significant stenosis, thereby improving overall patient care.

## 2. Methods and Materials

### 2.1. Methods

This section describes the methods employed in our research, emphasizing the effective utilization of convolutional neural networks (CNNs), a critical subset of artificial neural networks (ANNs) [12]. ANNs are the fundamental element in neural networks. Specifically, we focus on the comprehensive evaluation of distinguished CNN architectures: Visual Geometry Group (VGG), ResNet, and U-Net. To actualize our research methods, we embark on an explication of the rudimentary workings of ANNs. We start with a detailed comprehensive overview of the VGG and ResNet [13] and U-Net models, examining three distinct attributes and their notable contributions to deep learning.

#### 2.1.1. ResNet

Over the last few years, the sphere of deep learning (DL) has made major progress, such as medical image recognition and reinforcement learning. Amongst the miscellaneous neural network architectures that have been extended, ResNet [13] has garnered a considerable level of attention due to top-notch performance and accuracy. This revision investigates the selection of ResNet for identifying canal and foraminal stenosis, highlighting its architectural strengths and particular applications in this context. ResNet’s function in identifying canal and foramen l stenosis areas is pivotal due to its potential to vanquish ordinary DL challenges, such as missing gradient issue information. These complexes improve the network’s capability to achieve deep feature extraction and accuracy, which is crucial for accurately predicting and identifying canal and foramen l stenosis. Let x denote the input, y the output; $F\left(x,\left\{w_{i}\right\}\right)$ represents the residual mapping that needs to be learned. The function of a residual block in ResNet can be conveyed mathematically as

(1) $$y=F\left(x,\left\{w_{i}\right\}\right)+x$$

#### 2.1.2. VGG

It is a typical model of deep Convolutional Neural Network (CNN) design with a multitude of layers, and the acronym VGG [14] means Visual Geometry Group. The main contribution of this article is that, through the consecutive stacking of small convolutional filters (3 × 3), the CNN model can reach a deeper layer and acquire better accuracy and faster speed. Although this concept is simple, it is very important. There were still many questions and research studies on how to set the filter parameters. For example, AlexNet [15] used a considerable size (11 × 11), and GoogLeNet [16] also used different filter sizes. A prominent design theory of VGG is to fulfill high-accuracy performance through shallow and deep convolutional neural networks. Why does VGG [14] use 3 × 3 convolutional? Because this is the smallest size that can contain surrounding information. It is believed that the substitution of a larger convolutional with a more minor convolutional can improve the receptive field (it can also be said to increase the amount of information). In addition, using smaller convolutional can improve non-linearity while reducing parameters in contrast with large convolutional.

#### 2.1.3. U-Net

**Figure 1:**
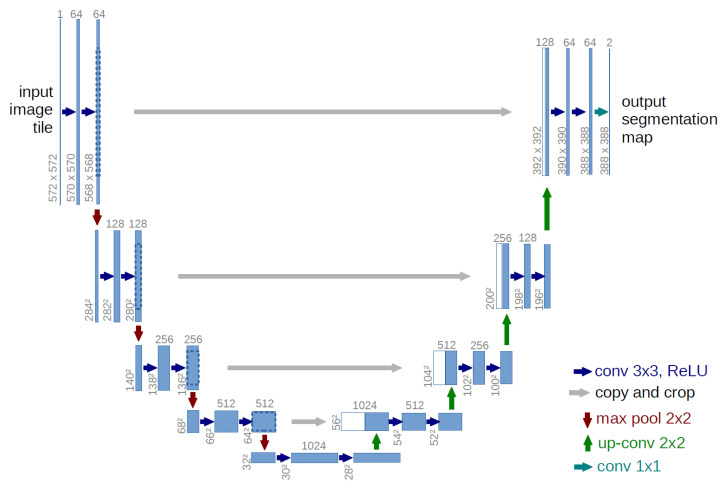
U-net architecture [17].

U-Net is a highly effective model for medical image segmentation tasks, such as separating cancerous tumors and segmenting biological cell nuclei. As illustrated in Figure 1, the U-Net architecture is symmetrical and U-shaped [17]. The model is mainly composed of a sequence of convolutional layers. The left segment, which forms the left side of the ‘U’ functions as the encoder, while the right segment, representing the right side of the ‘U’ acts as the decoder. A critical distinction between U-Net and a typical autoencoder [18] lies in the presence of skip connections in U-Net, which connect corresponding layers in the encoder and decoder.

**Figure 2:**
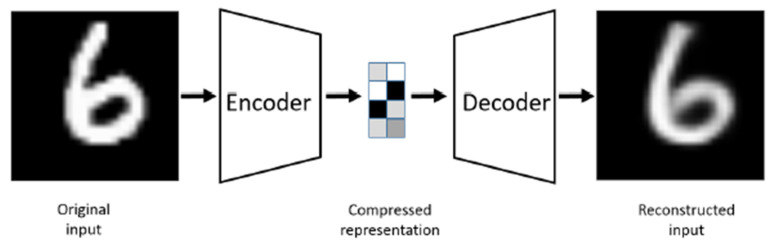
Autoencoder model [18].

The U-Net architecture closely resembles an autoencoder [18], with the critical difference being that U-Net’s input is an image, and its output is the segmented version of that image. Typically, the input and output images are of the same size. Figure 2 illustrates the autoencoder model for comparison. An autoencoder is a particularized neural network devised to rebuild its input as its output. For example, when processing an image of a handwritten digit, an autoencoder first encodes the image into a lower-dimensional latent representation. Then, it decodes this representation back into the original image. The autoencoder learns to compress the data and minimize reconstruction error, thereby capturing the essential features of the input.

#### 2.1.4. YOLO

YOLO (You Only Look Once) is a widely used and accessible Deep Learning (DL) algorithm for object detection in recent years to achieve fast and efficient object detection. DL is a critical branch of modern Artificial Intelligence (AI) and is a significant subfield of Machine Learning (ML). Within the expansive field of AI research, DL plays a pivotal role by enabling computers to perform highly complex tasks by learning intricate, layered data representations. Machine learning, which provides the foundational methods and technologies for allowing computers to learn and improve autonomously, is the cornerstone for advancing artificial intelligence.

**Figure 3:**
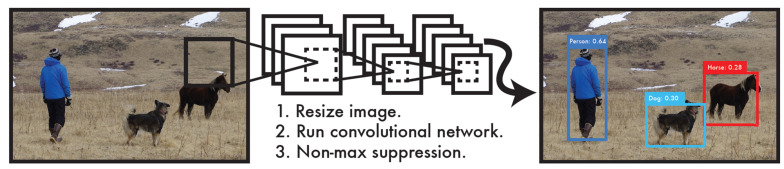
YOLO process diagram [19].

A notable feature of YOLO is its high-speed performance, making it particularly well-suited for real-time processing systems. At the core of its algorithm is a specialized improvement of the Convolutional Neural Network (CNN), as illustrated. The algorithm first scales the image and then inputs the scaled image into the Convolutional Neural Network. The YOLO process diagram is shown in Figure 3.

### 2.2. Material

#### 2.2.1. Spinal Canal Dataset

This spinal canal dataset comprises anonymized clinical Magnetic Resonance Imaging (MRI) scans from 515 patients experiencing back pain. Each patient may have one or more associated MRI studies. Each study consists of slices, or individual images, taken from either the sagittal or axial view, focusing on the lowest three vertebrae and the corresponding intervertebral discs (IVDs).

The axial view primarily captures slices of the lowest three IVDs, containing the disc between the last vertebra and the sacrum. The orientation of the slices for the lowest IVD follows the curve of the spine, while the slices for the other IVDs are typically parallel, forming block-like sections. Each IVD is represented by four to five slices, starting from the top and moving toward the bottom. The top and bottom slices often intersect the vertebrae, leaving one to three slices that cleanly capture the IVD without vertebral overlap. Each study usually consists of 12 to 15 axial view slices [20].

Given the complex nature of medical images, human errors, experience, and perception significantly impact the ground truth quality. This journal article presents the results of annotating lumbar spine Magnetic Resonance Imaging (MRI) images for automatic image segmentation. It introduces confidence and consistency metrics to assess the quality and variability of the resulting ground truth data [21].

#### 2.2.2. Intervertebral Foramen Dataset

The intervertebral foramen Magnetic Resonance Imaging (MRI) dataset utilized in this study was sourced from the Radiopaedia website, a non-profit, international collaborative radiology education platform. This dataset comprises normal sagittal spine MRI scans from roughly 40 subjects of varying ages and sex. It includes both T1-weighted and T2-weighted MRI scans. Each patient’s sagittal spine MRI dataset comprises approximately 15 to 20 sagittal slices. However, not each slice intersects with this intervertebral foramen, so we manually choose the sagittal sections that precisely capture the foramen [22,23].

#### 2.2.3. Intervertebral Foramen and Spinal Canal Flowchart

The spinal canal and intervertebral foramen are distinct anatomical structures with different functional roles: the spinal canal primarily protects the spinal cord, while the foramen serves as a passage for nerve roots. As a result, separate datasets are necessary to ensure focused and accurate segmentation of each structure. The spinal canal dataset includes MRI scans from 515 patients, allowing for a comprehensive analysis of its features. In contrast, the intervertebral foramen dataset, sourced from an educational radiology platform (Radiopaedia), contains scans from 40 subjects. These datasets, drawn from different populations, reflect each region’s unique anatomical and imaging characteristics. Furthermore, the imaging modalities vary, with spinal canal imaging using axial slices and intervertebral foramen imaging relying on sagittal views, necessitating distinct annotation approaches. Using different patient populations and imaging parameters enhances the accuracy of the deep learning models for segmenting the spinal canal and intervertebral foramen, each of which requires tailored dataset diversity and imaging resolution. Moreover, it also helps assess our models’ robustness across varied demographic and pathological conditions, thus enhancing the applicability of our findings in natural clinical settings.

We used the Computer Vision Annotation Tool (CVAT) to label the spinal canal, facet joint, and intervertebral disc in the spinal Magnetic Resonance Imaging (MRI) data. The marking format is a segmentation mask. The segmentation mask will be used as the ground truth, which is essential for training and validating the spinal canal segmentation model.

For image preprocessing, our study utilizes a spinal canal MRI dataset and manually annotates segmentation masks of the spinal canal area to train this segmentation model. The dataset includes 515 subjects, each with an original spinal canal MRI image and a corresponding ground truth annotation. A total of 300 MRI images were selected for the training dataset, 100 for the test dataset, and the remaining 115 images were the validation dataset.

**Figure 4:**
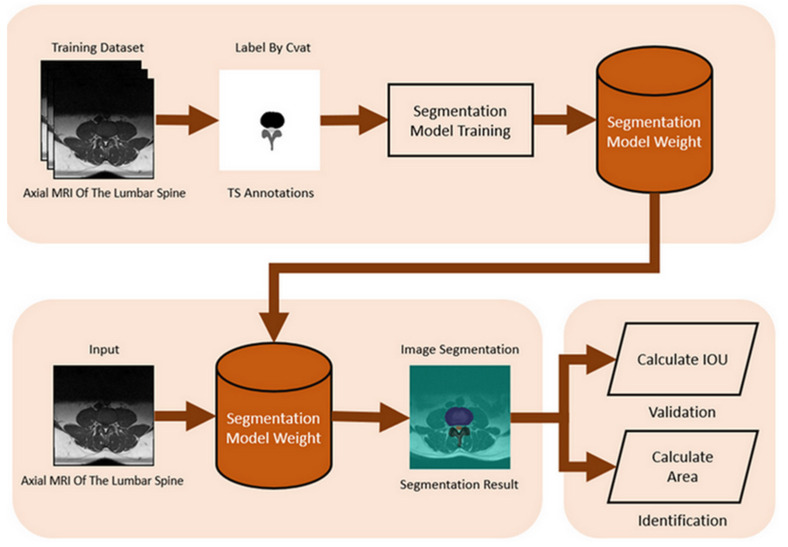
The flowchart of spinal canal verification and identification.

We use libraries, tensorflow, and computer vision. TensorFlow is an open-source software library for machine learning for various perception and program language understanding tasks. This case needs to identify three portions: the facet joints, intervertebral disc, and spinal canal, so this model’s hyperparameters are set to width = 320 and height = 320. Input the image to be tested, apply the trained segmentation model to identify the spinal canal area, and store the recognition result as a segmentation mask. The spinal canal verification and identification of the flowchart is shown in Figure 4.

**Figure 5:**
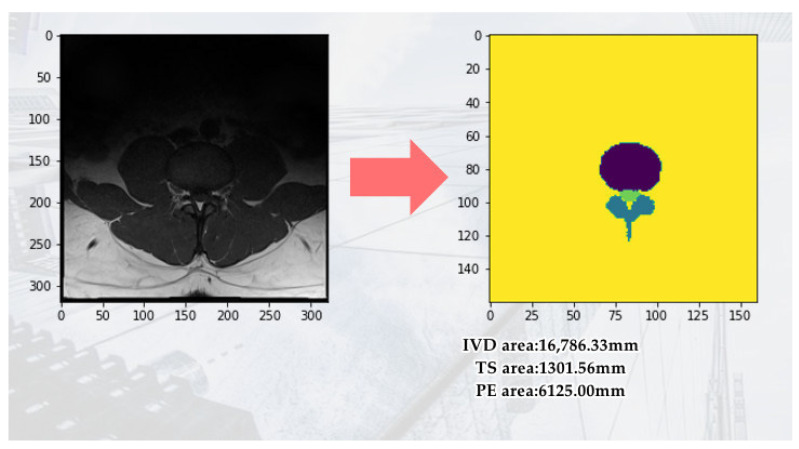
Use FOV to calculate the converted area (mm^2^).

An important parameter of an MRI image is the Field of View (FOV). FOV refers to the imaging range set during an MRI scan, representing the actual length and width of the image. Our study uses the FOV value from the spinal canal MRI image to convert the pixel dimensions in the identification results into actual size (mm^2^). First, we calculate the number of pixels corresponding to the spinal canal area in the identification result. Then, by multiplying the number of pixels by the genuine size of each pixel, we acquire the cross-sectional area of the spinal canal for the subject. The FOV application to calculate the converted area (mm^2^) is shown in Figure 5. This approach starts by bringing to bear semantic segmentation to the MRI images to identify three regions of interest: The Posterior Element (PE), Intervertebral Disc (IVD), and Thecal Sac (TS).

The labeling work is completed using LabelImg, an image labeling tool that is widely used for object labeling. Image preprocessing for the intervertebral foramen dataset, which includes 40 different subjects, involves handling variations in the aspect size of each MRI image. We resize each image to 416 × 416 pixels to standardize the dataset. Additionally, we apply data augmentation to the dataset. Proper data augmentation helps prevent overfitting during training. Specifically, we enhance the original 80 MRI images by generating an additional 80 images through random rotations, hue adjustments, and brightness modifications.

**Figure 6:**
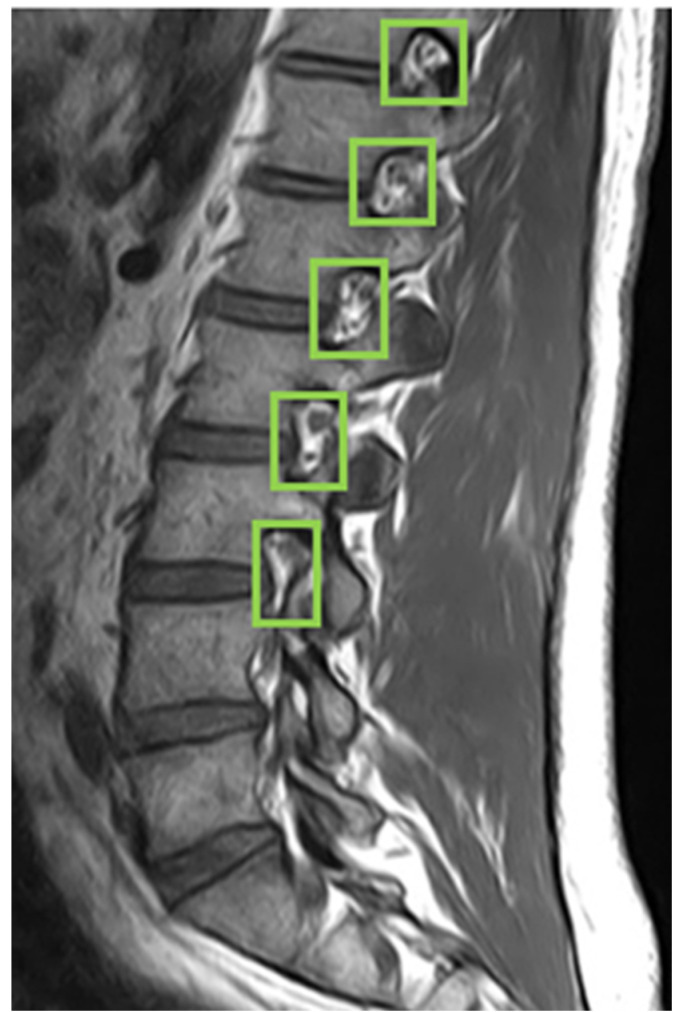
Use LabelImg tool to annotate intervertebral foramen MRI image as green boxes.

First, we used LabelImg software version v1.8.1 (https://github.com/HumanSignal/labelImg/releases, accessed on 11 February 2023) to delineate the rectangular region of the intervertebral foramen. The annotations were made in YOLO format, with the intervertebral foramen as the sole labeled class. Figure 6 shows an example of a light-green-colored annotated intervertebral foramen MRI image.

We allocated 75% of the annotated dataset for training and 25% for validation. The model was trained using YOLOv4 with a batch size of 64, a learning rate of 0.00261, and a maximum of 8000 batches. YOLO (You Only Look Once) is a one-stage object detection algorithm originally released and developed by Joseph Redmon in 2015. YOLOv4, released in 2020, is known for its speed and high accuracy, making it one of the leading object detection algorithms.

This study used CVAT software version v2.3.0 (https://docs.cvat.ai/v2.3.0/docs/manual/, accessed on 11 February 2023) to annotate the intervertebral foramen in the MRI images, with the annotations in the form of segmentation masks. These segmentation masks serve as the ground truth, essential for training and validating the intervertebral foramen segmentation model.

The process of intervertebral foramen object detection begins by inputting the MRI image and using the trained YOLOv4 model to detect the intervertebral foramen within it. Once the intervertebral foramen is identified, the region containing it is cropped into independent rectangular sub-images. These sub-images are then processed using a trained segmentation model to identify the intervertebral foramen area in each one accurately. The recognition results are then stored as segmentation masks.

**Figure 7:**
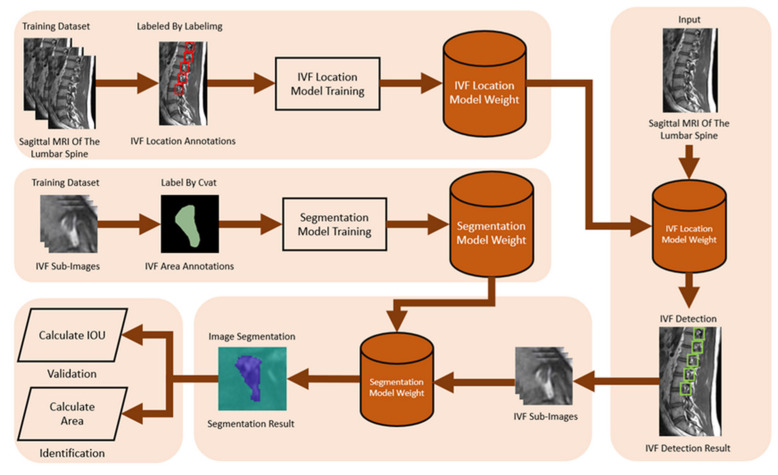
The flowchart for intervertebral foramen verification and identification.

In morphology, image noise is commonly present in the results identified by the segmentation model, which can affect the accuracy of area calculations. To address this, we applied opening and Gaussian blur [24] operations to refine the identified results. We then used the same method to calculate the IoU for the spinal canal to determine the intervertebral foramen IoU, which indicates the model’s accuracy. Similarly, we applied the spinal canal area calculation method to determine the intervertebral foramen’s actual area, measured in square millimeters (mm^2^). The flowchart for intervertebral foramen verification and identification is shown in Figure 7.

## 3. Experimental and Hyperparameter Settings

### 3.1. Experimental

We used the public spinal canal and intervertebral foramen dataset as the training data in the experiments. The model used a hardware setup featuring a GeForce RTX 2070 WINDFORCE 8 G GPU and 32 GB of RAM (NVIDIA, Santa Clara, CA, USA). The software environment consisted of Windows 10 as the operating system, along with Python 3.8 (https://www.python.org/downloads/release/python-380/, accessed on 3 March 2023) and CUDA 10.2 (https://developer.nvidia.com/cuda-10.2-download-archive, accessed on 1 December 2022). The hardware experimental environment specification list is shown in Table 1.

### 3.2. HyperParameter Settings

When training neural networks, adjusting hyperparameters can lead to better results. However, image data labeling is one of the most critical and time-consuming stages in this process, particularly when human resources are limited. In such scenarios, obtaining an immense number of labeled images for training in a short period can be challenging, often resulting in a dataset that is insufficiently sized. Consequently, the time efficiency of data labeling becomes an important issue.

To solve this problem, we employed data augmentation techniques in our research. This approach allows us to generate numerous variant images from a limited set of labeled images, effectively expanding the training dataset without incurring additional manual labeling costs. This method improves the efficiency of data labeling and enhances the model’s learning capability and generalization performance. The YOLOv4 hyperparameter setup is shown in Table 2.

### 3.3. Evaluation Metrics

Intersection over Union (IoU) is a standard metric used to measure the accuracy of detecting corresponding objects within a specific dataset. The IoU indicator will help us fully understand the model’s relevance and guide us to further optimization. IoU is computed by dividing the intersection of the predicted result with the ground truth by their union.

(2) $$IoU=\frac{area(A\cap B)}{area(A\cup B)}$$

In the formulas, True Positives (TP) refers to the number of objects correctly identified, False Positives (FP) denotes the number of objects incorrectly identified as belonging to a different class, and False Negatives (FN) represents the number of objects from the target class that were incorrectly classified as belonging to another class. Therefore, TP + FP represents the total number of objects predicted by the model, while TP + FN represents the actual number of objects in the target class.

The Average Precision (*AP*) was used to evaluate the proposed method’s model detection and segmentation performance. All the *AP* values of all categories were also averaged to output mean Average Precision (*mAP*). The calculation Formula (3) for *mAP* is as follows:

(3) $$mAP=\frac{\sum_{n=1}^{N}{AP}_{n}}{N}$$

## 4. Results

### 4.1. Spinal Canal Identification

#### Training Different Segmentation Models Based on Image Category

The MRI images of the subject’s spinal canal include D3, D4, and D5, with each section imaged using two weighting methods, T1 and T2, resulting in a total of six images. The spinal canal, intervertebral discs, and facet joints differ slightly in shape and size across these six images. To prevent these differences from affecting segmentation performance, we trained separate segmentation models for each MRI image of the spinal canal. This approach aims to minimize the impact of variations between different images on segmentation accuracy.

**Figure 8:**
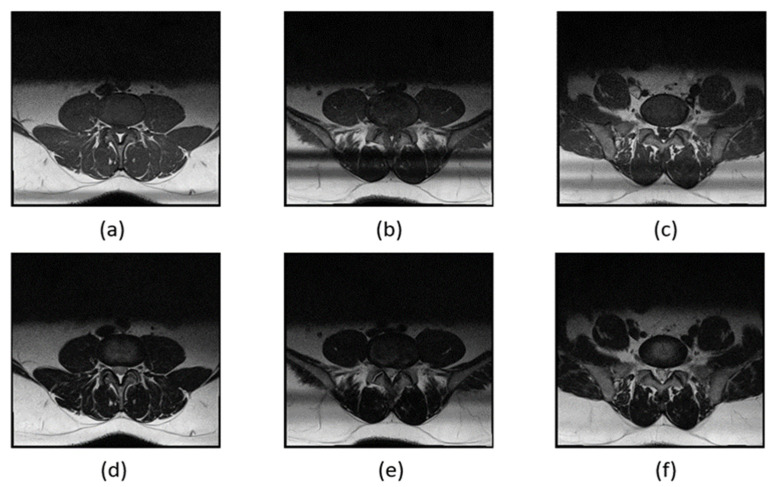
Spinal canal in different parts in T1–T2. From Figure 8, it can be observed that there are slight differences in pictures (**a**–**f**). (**a**) The facet joints of the spinal canal in the MRI images of L3 and L4 are more obvious, and the L5 site is connected to the S1 site; (**b**) therefore, the shape of the spinal canal in the MRI image will be more special. (**c**) T1 and T2 weighting also had a significant influence on the image. (**d**) T1 weighting will enhance the fat part in the MRI image; (**e**) T2 weighting will strengthen the watery part in the image, and thus the spinal canal part in the T1 weighted MRI image appears to be completely black, (**f**) whereas in T2-weighted MRI images, the spinal canal is lighter and some white nerves can be observed.

Noticeable differences were found between images (a) and (f). In the MRI images of L3 and L4, the facet joints of the spinal canal are more pronounced, while the L5 segment is connected to the S1 segment, giving the spinal canal a distinct shape. T1 and T2 weighting also have a significant impact on the image appearance. T1 weighting enhances the fat tissues in the MRI image, making the spinal canal appear entirely black, whereas T2 weighting highlights the watery tissues, resulting in a lighter spinal canal where some white nerves are visible. The spinal canal in different sections using T1 and T2 weighting is illustrated in Figure 8.

### 4.2. Model Comparison

We utilized ResNet50 and VGG16 as the encoding architectures, combined with U-Net and SegNet [25] as the decoding architectures, to form four segmentation models: VGG_U-Net, VGG_SegNet, ResNet_SegNet, and ResNet_U-Net. The performance of these models was evaluated using the mean Intersection over Union (IoU) and the standard deviation of IoU (IoU Std) as metrics. The model with the highest mean IoU and the lowest IoU Std was selected as the best segmentation model.

This compares the mean IoU and IoU standard deviation of the four segmentation models: VGG_U-Net, VGG_SegNet, ResNet_U-Net, and ResNet_SegNet. Among them, ResNet_U-Net has the best performance. Overall, it can be found that Resnet50 performs better than VGG16 in the case of the spinal canal. A comprehensive comparison is shown in Table 3.

### 4.3. Intervertebral Foramen Identification

**Figure 9:**
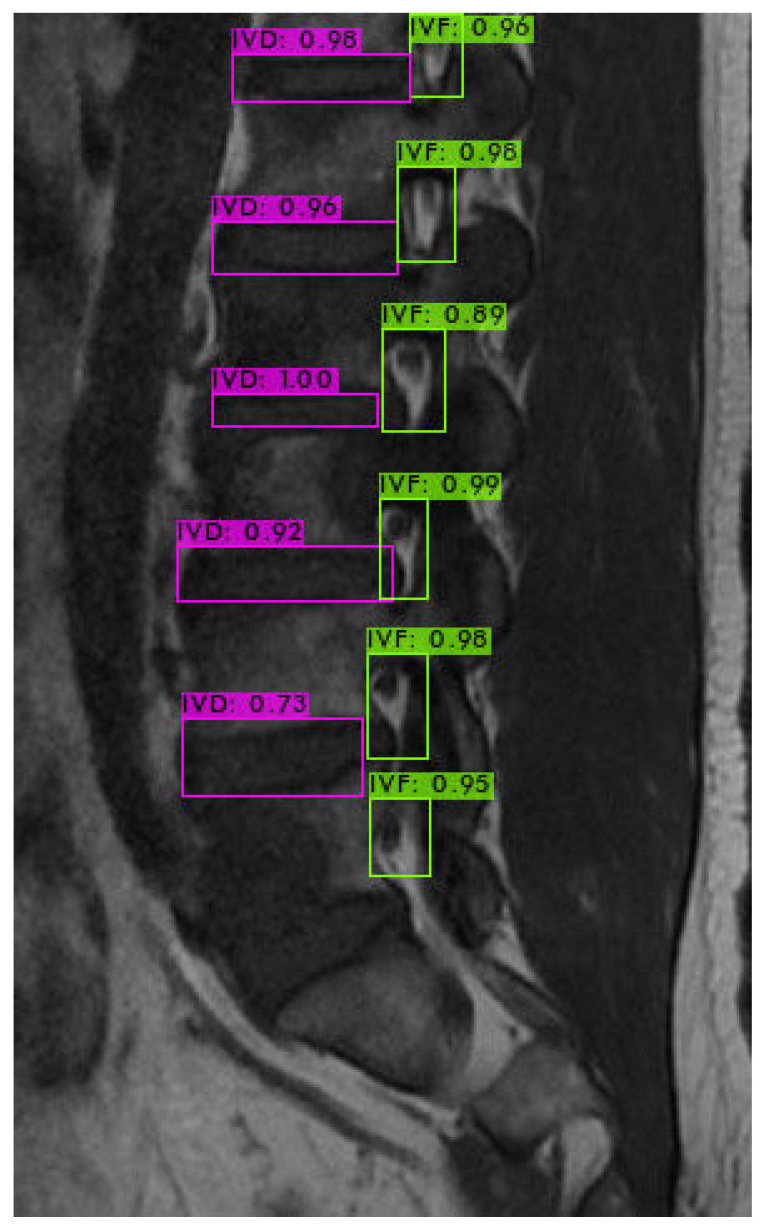
YOLOv4 object detection.

For identifying the area of the intervertebral foramen (IVF) and intervertebral disc (IVD), we first employ the YOLOv4 object detection model to detect the intervertebral foramen in MRI images shown in Figure 9. The detected foramen is then cropped into sub-images, which are further processed using DL methods to segment the areas of the IVF and IVD within these sub-images. After segmentation, the number of pixels occupied by the intervertebral foramen in the MRI dataset is analyzed. Finally, by using the Field of View (FOV), detailed information from the MRI dataset, this pixel count is converted into an actual area measurement in square millimeters.

The experiment reveals that during the training process of the model using YOLOv4, various performance indicators are displayed, including the changes in loss and mean Average Precision (*mAP*). The following is an analysis of the training process: The blue line (Loss) represents the model’s loss value during training. Initially, the loss value decreases rapidly, indicating that the model learns features from the images effectively. As the number of iterations rises, the loss of numerical value continues to decrease steadily and gradually stabilizes.

**Figure 10:**
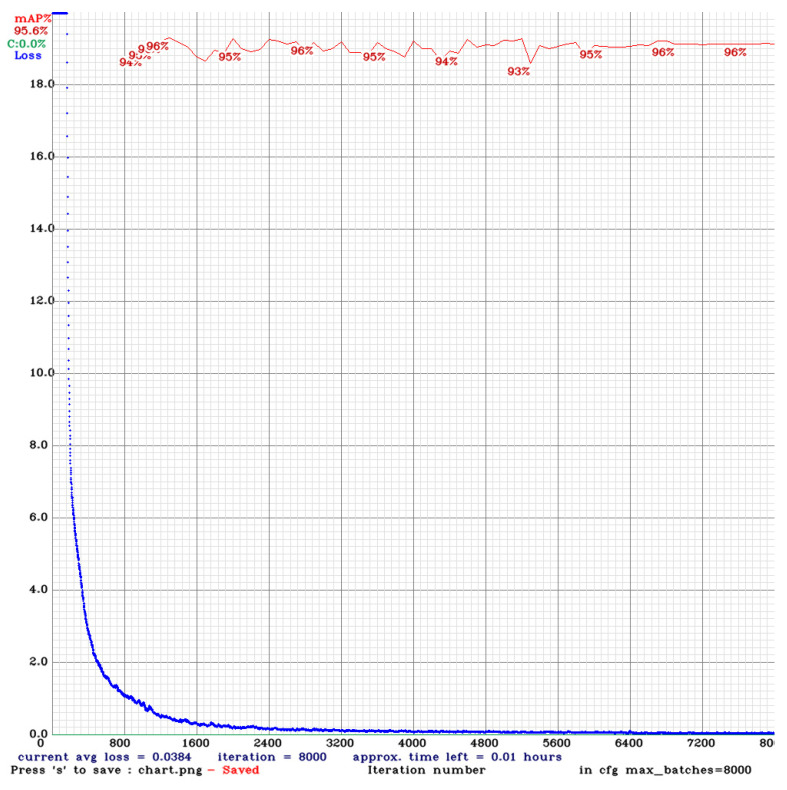
YOLOv4 training process.

As shown in Figure 10 about the YOLOv4 training process, the initial *mAP* value is 94%. As the number of training iterations elevates, the loss diminishes, and the *mAP* rises, reaching a maximum of 96%, around 1300 iterations. However, as training continues, overfitting begins to occur, where the model becomes overly specialized to the training image dataset, making it challenging to predict or differentiate new, unseen data accurately. This leads to a slight decrease in *mAP*. Despite this, the *mAP* value stabilized at 96% between 6000 and 8000 iterations, with the final *mAP* recorded at 95.6%.

### 4.4. Assessment of Models and Morphological Processing Techniques

Similarly to the comparison of different models for spinal canal identification, we employed VGG16 and ResNet50 as the encoding architectures and U-Net and SegNet as the decoding architectures, forming four segmentation models: VGG_U-Net, VGG_SegNet, ResNet_U-Net, and ResNet_SegNet. We evaluated the performance of these models using the mean IoU and IoU standard deviation (IoU Std) indicators. The model with the highest average IoU and the lowest IoU standard deviation was selected as the best segmentation model based on the experimental results.

The ResNet50-U-Net model achieved the highest IoU mean value and the lowest IoU Std value. Table 4 compares the IoU mean and IoU Std values of the four segmentation models—VGG_U-Net, VGG_SegNet, ResNet50_U-Net, and ResNet_SegNet—without morphology processing. Consistent with the results of spinal canal identification, ResNet50-U-Net demonstrated the best performance.

The ResNet50-U-Net model achieved the highest IoU mean value and the lowest IoU Std value. Table 4 compares the IoU mean and IoU Std values of the four segmentation models—VGG_U-Net, VGG_SegNet, ResNet50_U-Net, and ResNet_SegNet—after applying morphology processing. The identification results are consistent with those in Table 5. It can be observed that after applying morphology processing, the IoU mean for VGG_U-Net and ResNet50-U-Net, which use U-Net as the decoding model, improved, while the IoU Std remained largely unaffected. Conversely, the IoU mean for VGG_SegNet and ResNet50_SegNet, which use SegNet as the decoding model, decreased, along with a reduction in IoU Std.

## 5. Discussion

With increasing age in the population, the incidence of spinal stenosis is expected to slowly increase, with approximately 266 million people suffering from lumbar degenerative diseases each year. This trend underscores the growing importance of developing quick, convenient, and standardized methods for detecting spinal stenosis, which has emerged as a key challenge in the medical realm.

Earlier investigations have utilized deep learning techniques to identify spinal stenosis; their indicators often lack precision, relying on coarse metrics such as four-point scales or dichotomous classifications to represent severity. In this study, we first used YOLO to locate intervertebral discs (IVD) and intervertebral foramina (IVF) and then utilized the area as more refined indicators to assess spinal stenosis. Our approach significantly achieves the 95.6% mean Average Precision (*mAP*) of detection.

Using areas as indices for assessing spinal stenosis enhances patients’ understanding of symptom severity and facilitates hospital data collection and standardization. On top of that, by introducing new models and indicators, this paper found that applying dilation and erosion skills to address image noise produced precise image results. The two models, Resnet50 and U-Net, that we employed to discern the spinal canal and intervertebral foramen achieved IoU scores of 79.11 and 80.89, respectively, indicating reliable performances. Although there is still some way to match mature object recognition models, this study has established a precedent for using areas as indicators in assessing spinal stenosis.

Looking ahead, more reliable indicators or models can be developed, and code for identifying MRI images can be released. The concepts and models proposed in this paper could be adapted to imaging other anatomical regions.

In conclusion, spinal canal MRI images are captured using two weighting methods, T1 and T2, across three sections, L3, L4, and L5, resulting in six images. The segmentation model was trained separately for each spinal canal MRI image, with the ResNet50-U-Net model demonstrating the best performance, achieving an IoU as high as 80.89% (noting that the standard benchmark for a reasonable recognition rate is an IoU of 0.5 or higher). Overall, the results indicate that ResNet50 outperforms VGG16 in spinal canal identification.

To identify the intervertebral foramen area, we will compare the results obtained with and without erosion and dilation processing and evaluate the influence of these image-processing techniques on the identification outcomes. Among the approaches that exclude erosion and dilation, the ResNet50-U-Net model demonstrates the best performance. Using U-Net as the decoding model improves the IoU mean for intervertebral foramen identification when applying morphological processing.

While the models we used (such as ResNet, VGG, U-Net, and YOLO) are not the most recent state-of-the-art models, they still hold significant value in our research for several reasons: their reliability and stability, which have been extensively validated over many years; the rich literature support available due to their widespread use; their computational efficiency, which is crucial for processing large volumes of medical imaging data; their proven suitability for our specific spinal MRI dataset; their interpretability, which is particularly important in medical applications; and the ease of benchmark comparison with other studies. Our research focuses on applying these well-validated models to spinal MRI analysis and optimizing their performance through data augmentation, hyperparameter tuning, and other methods. Our results demonstrate that these models perform excellently in our task, achieving high accuracy. These time-tested models provided us with a reliable, efficient, and interpretable solution for this research. In future studies, we plan to explore and evaluate the performance of newer, more advanced models in this domain.

## 6. Conclusions

Our study is the first to use deep learning mixed with computer vision to identify spinal stenosis, using Intersection over Union (IoU) and mean Average Precision (*mAP*) as verification indicators. The approach involves segmenting the relevant anatomical structures and then calculating their areas. Unlike previous studies that categorized symptoms into broad two-part or four-part classifications, this research provides a more explicit and objective assessment of the degree of spinal stenosis. Additionally, by employing techniques such as morphological processing, the study significantly improves the average IoU and *mAP* for intervertebral foramen identification.

## Figures and Tables

**Table 1:** Hardware experimental environment specification list.

Experimental Environment	Specifications
Operating System	Windows 10
CPU	AMD Ryzen 7 2700X Eight-Core Processor 3.70 GHz
GPU	GeForce RTX 2070 WINDFORCE 8G
CUDA Version	10.2
RAM	32.0 GB
Framework	Darknet
Python	3.8

**Table 2:** YOLOv4 hyperparameters setup.

Hyperparameter Name	Value
learning rate	0.00261
policy	steps
max_batches	8000
activation	leaky
momentum	0.9
decay	0.0005
angle	180
saturation	1.5
exposure	1.5
hue	0.1
gaussian noise	20

**Table 3:** The comparison of different model’s IoU mean and IoU Std value.

S.No	Encode Model	Decode Model	IoU Mean	IoU Std
1	VGG16	U-net	73.94	9.54
2	VGG16	Segnet	66.94	10.38
3	Resnet50	U-net	77.4	8.77
4	Resnet50	Segnet	68.61	12.58

**Table 4:** This table represents the comparison the four different segmentation of model’s IoU mean and IoU Std value.

S.No	Encode Model	Decode Model	IoU Mean	IoU Std
1	VGG16	U-Net	61.19	10.85
2	VGG16	Segnet	48.11	12.28
3	Resnet50	U-Net	79.11	9.40
4	Resnet50	Segnet	50.47	14.42

**Table 5:** This table represents the comparison of the four different segmentation within after morphology processing model’s IoU mean and IoU Std value.

S.No	Encode Model	Decode Model	IoU Mean	IoU Std
1	VGG16	U-Net	64.97	10.85
2	VGG16	Segnet	45.26	8.99
3	Resnet50	U-Net	80.89	9.44
4	Resnet50	Segnet	43.09	10.60

## Data Availability

Data supporting this study are openly available from Radiopaedia at https://radiopaedia.org/cases/47857 (accessed 10 September 2023). https://radiopaedia.org/cases/40976 (accessed 15 November 2015) and Lumbar Spine MRI Dataset at http://dx.doi.org/10.17632/zbf6b4pttk.2 (accessed 20 November 2023).
